# INCREASED C3 IN THE AGING BRAIN PROMOTES INFLAMMATORY TRANSITION IN ENDOTHELIAL CELLS

**DOI:** 10.1093/geroni/igz038.3093

**Published:** 2019-11-08

**Authors:** Nicholas E Propson, Alexandra Litvinchuk, Ethan R Roy, Bianca Contreras, Wei Cao, Hui Zheng

**Affiliations:** Baylor College of Medicine, Houston, Texas, United States

## Abstract

Innate immunity has been implicated in normal aging, and age-related disease. The connection between age-related neuroinflammation and change in brain vasculature prior to disease onset remains poorly understood. The complement pathway is an established mediator of neuroinflammation, and increased complement C3 is seen in the aging brain. Thus, we asked whether C3 can promote changes in brain vasculature. We found age dependent increase of brain C3 levels in C57BL/6J mice. Furthermore, we found an increase in expression of adhesion molecule VCAM-1 in endothelial cells (ECs) of the cortex and hippocampus, which was rescued in aged C3a receptor null (C3ar1-/-) mice and aged C3a receptor (C3aR) antagonist treated mice. We confirmed these results by qPCR analysis for Vcam1 in sorted ECs. Human brain microvascular endothelial cells (HBMECs) treated with C3a showed increased expression of VCAM-1, but not other adhesion molecules. Sorted ECs from C3ar1-/- mice challenged with LPS confirmed these findings. Furthermore, C3aR signaling in ECs showed increased blood-brain barrier (BBB) permeability using trans-endothelial electrical resistance (TEER), and BBB impermeable dye injections. HBMECs treated with C3a revealed mis-localization of VE-Cadherin, followed by reduction in protein level when analyzed by immunofluorescence, which promotes increased barrier permeability. As a functional consequence of VCAM-1 expression and increased BBB permeability we found aged mouse brains have increased peripheral lymphocyte (CD45+/CD11b-) infiltration, which was reduced in a C3aR dependent manner. In conclusion, our work suggests there is a strong relationship between C3 expression and vascular C3aR contributing to a functional transition in endothelial cells during aging.

